# 180 Beyond Days of Therapy: The Anaerobic Activity Index (AAI) Strongly Correlates with Shannon Diversity and Pathogen Expansion

**DOI:** 10.1017/ash.2026.10726

**Published:** 2026-06-23

**Authors:** Megan Sutton, Grace Lee, Andrea Salinas, Ashley Marin

**Affiliations:** 1 Louisiana Dept of Health; 2 LOUISIANA DEPARTMENT OF HEALTH - OFFICE OF PUBLIC HEALTH; 3 Louisiana Department of Health

## Abstract

**Background:** Carbapenem-resistant Acinetobacter baumannii (CRAB) is an opportunistic organism that can cause serious infections that are difficult to treat. CRAB often possesses concerning resistance mechanisms, including carbapenemase enzymes, which can spread resistance through mobile genetic elements. Prevention-driven point prevalence surveys (PPSs), a strategy outlined in the Centers for Disease Control and Prevention’s multidrug-resistant (MDRO) prevention guidance, can enable early identification of colonized patients and inform targeted infection control measures to mitigate transmission. From 2024-2025, the Louisiana Office of Public Health partnered with a long-term acute care hospital (LTACH) to implement prevention-driven PPSs for CRAB. **Methods:** LTACHs were targeted due to the increased risk of MDRO colonization among high-acuity patients admitted for prolonged periods. A webinar was held for LTACH facilities in a region of the state with a high CRAB burden, and one facility agreed to participate. The PPSs were conducted quarterly for one year. Testing was performed by the Southeast Antimicrobial Resistance Laboratory Network (ARLN) and included organism confirmation, real-time polymerase chain reaction to detect carbapenemase genes, antimicrobial susceptibility testing, and whole genome sequencing (WGS). Methods are described on ARLN's website. Genetic relatedness and pairwise single nucleotide polymorphism (SNP) differences among isolates were analyzed using the National Center for Biotechnology Information Pathogen Detection Isolate Browser. **Result:** From February 2024 to January 2025, 121 specimens were collected from 87 patients. Screening sites included axilla-groin (71%), wounds (20%), and rectum (9%). Five percent (4/87) of the patients were found to be colonized with CRAB. These isolates were resistant to all carbapenems tested (meropenem, doripenem, and imipenem), produced OXA-23 or OXA-24/40 carbapenemases, and two of the isolates were pan-resistant. Three CRAB patients were identified within the same SNP cluster and were closely related, averaging 12 SNP differences. One CRAB patient was also colonized with Stenotrophomonas maltophilia. Additionally, 4 different patients (5%) tested positive for Pseudomonas aeruginosa; 3 were carbapenem-resistant, and of these, 1 was pan-resistant. Colonized patients exhibited typical risk factors for MDRO colonization, including underlying conditions, wounds, recent antimicrobial exposure, and indwelling device use. **Conclusion:** Screenings enabled routine assessment of CRAB colonization burden. PPSs identified colonized patients who may have otherwise gone unrecognized, allowing for timely implementation of infection control actions. WGS detected a previously-unknown cluster, indicating transmission within healthcare facilities. These findings align with and support current MDRO containment guidance and underscore the importance of conducting screenings in high-risk healthcare settings to reduce MDRO exposure among vulnerable patients.

